# Qishen Yiqi Dropping Pills Protect Against Myocardial Infarction in Mice via Activating SIRT3/FOXO3a Signaling Pathway

**DOI:** 10.3390/ph19030489

**Published:** 2026-03-16

**Authors:** Canran Wang, Da Wo, Yi Huang, Xiyao Zhang, Celiang Wu, En Ma, Yuhang Gong, Jinxiao Chen, Weidong Zhu, Dan-ni Ren

**Affiliations:** Academy of Integrative Medicine, College of Integrative Medicine, Fujian Key Laboratory of Integrative Medicine on Geriatric, Fujian University of Traditional Chinese Medicine, Fuzhou 350122, China; 18437850478@163.com (C.W.); dwo_work@126.com (D.W.); 19204900535@163.com (Y.H.); 13400228160@163.com (X.Z.); 15821726396@163.com (C.W.); 18217497624@163.com (E.M.); yuhang_galal@163.com (Y.G.); shangtang002002@163.com (J.C.); wzhu@tongji.edu.cn (W.Z.)

**Keywords:** Qishen Yiqi dropping pills, myocardial infarction, oxidative stress, SIRT3/FOXO3a signaling pathway

## Abstract

**Background**: Myocardial infarction (MI) is the leading cause of morbidity and mortality globally. A major pathological progression of MI is the excess generation of reactive oxygen species (ROS), which results in oxidative stress and damage to the ischemic heart. Because damage to the myocardium is irreversible, the development of new therapeutic agents that can decrease the degree of ischemic damage following MI is crucial. The traditional Chinese medicine formulation, Qishen Yiqi dropping pills (QSYQ), has been clinically used in the treatment of various cardiovascular diseases; however, the precise mechanisms underlying its therapeutic effects remain unelucidated. **Methods**: In this study, we established murine models of MI via coronary artery ligation to investigate the protective effects and mechanisms of QSYQ following MI. **Results**: The administration of QSYQ significantly improved cardiac function, reduced infarct size, and attenuated ventricular remodeling in mice that underwent MI. Moreover, MI-induced oxidative stress and downregulated levels of antioxidant enzymes were prevented in mice administered QSYQ via upregulating the SIRT3/FOXO3a signaling pathway. Importantly, pretreatment with a selective SIRT3 inhibitor 3-TYP abolished the cardioprotective effects of QSYQ. **Conclusions**: Our findings elucidate the role and mechanism of QSYQ in protecting against oxidative damage and restoring redox homeostasis following myocardial infarction. This study provides support for the therapeutic potential of QSYQ in the clinical treatment of myocardial ischemic diseases.

## 1. Introduction

Myocardial infarction (MI) is the most common outcome of ischemic heart diseases and remains the leading cause of mortality globally [1,2,3]. The acute restriction of coronary blood flow, commonly caused by plaque buildup, triggers the widespread death of surrounding cardiomyocytes, resulting in severe cardiac dysfunction [4]. Although prompt reperfusion therapy can restore coronary blood flow, damage to the myocardium is irreversible because the heart lacks an endogenous repair mechanism [5,6]. Therefore, novel therapeutic strategies that can effectively mitigate myocardial ischemic injury is of paramount clinical significance for improving patient outcomes.

The pathophysiology of MI is a complex process driven by the production of reactive oxygen species (ROS), which contributes to various adverse outcomes such as DNA damage and cardiomyocyte death [7,8]. The generation and elimination of intracellular ROS are tightly regulated by pro-oxidant and antioxidant systems in a state of redox homeostasis [9]. However, the incidence of pathological MI causes the excessive production of ROS production, which disrupts this redox equilibrium [10]. Therefore, if the production of endogenous antioxidant enzymes including catalase and superoxide dismutase (SOD) cannot be enhanced following MI, the disruption to redox homeostasis would result in the exacerbation of myocardial damage [11]. Several signaling pathways are involved in the activation of the antioxidant defense system, among which the SIRT3/FOXO3a pathway plays a key role in the modulation of antioxidant enzymes in the pathological setting [12]. Hence, drugs that can enhance endogenous SIRT3/FOXO3a signaling may constitute a promising therapeutic strategy in ischemic myocardial injury.

Qishen Yiqi dropping pills (QSYQ) is a traditional Chinese medicine formulation that is composed of *Astragalus membranaceus* (Huangqi), *Salvia miltiorrhiza* (Danshen), *Panax notoginseng* (Sanqi), and *Dalbergia odorifera* (Jiangxiang) and is used in the clinical management of various cardiac diseases [13,14,15]. Various bioactive components contained in QSYQ, such as astragaloside IV and tanshinones, have been demonstrated to exert multi-target cardioprotective effects [16,17]. Studies have demonstrated that QSYQ exhibits a strong protective effect in ischemic heart diseases, notably in animal models of MI [14,18,19]. However, the precise molecular mechanisms of QSYQ, particularly in the regulation of MI-induced oxidative stress, remain largely unexplored. In this study, we aimed to investigate the cardioprotective effects and underlying mechanisms of QSYQ in acute myocardial ischemic injury via the activation of the SIRT3/FOXO3a-regulated antioxidant defense system.

## 2. Results

### 2.1. High-Performance Liquid Chromatography–Mass Spectrometry (HPLC-MS/MS) Analysis of QSYQ Powder

**Figure 1:**
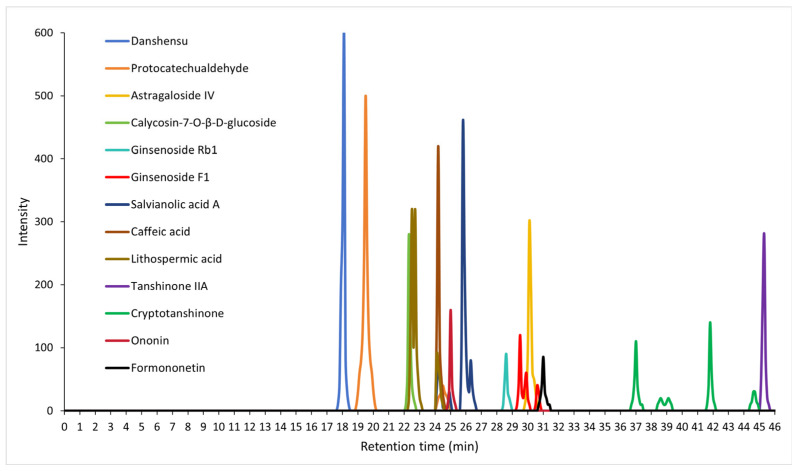
HPLC chemoprofiles of the top bioactive compounds in QSYQ powder. Bioactive compounds are shown according to their retention times and corresponding intensity peaks.

We used HPLC-MS/MS and identified the ratio of each of the four components contained in Qishen Yiqi dropping pills (QSYQ) as follows: *Astragalus membranaceus* (Fisch.)—Huangqi 31–32%, *Salvia miltiorrhiza* Bunge 61–62%—Danshen, *Panax notoginseng* (Burk.) F.H.Chen—Sanqi 6–7%, and *Dalbergia odorifera* T.Chen—Jiangxiang 0.5–1.5%. Furthermore, we elucidated the top bioactive compound structures contained in the QSYQ extract based on their retention times and intensity peaks (as well as the component from which each bioactive compound was derived), quantified using calibration curves of corresponding chemical standards, including: danshensu (Danshen, main peak at 18.043 min), protocatechualdehyde (Danshen, main peak at 19.516 min), salvianolic acid A (Danshen, main peak at 25.804 min), caffeic acid (Danshen and Huangqi, main peak at 24.167 min), lithospermic acid (Danshen, main peak at 22.706 min), astragaloside IV (Huangqi, main peak at 30.139 min), calycosin-7-O-β-D-glucoside (Huangqi, main peak at 22.308 min), tanshinone IIA (Danshen, main peak at 45.195 min), cryptotanshinone (Danshen, main peak at 41.801 min), ononin (Huangqi, main peak at 24.973 min), ginsenoside Rb1 (Sanqi, main peak at 28.571 min), ginsenoside F1 (Sanqi, main peak at 29.489), and formononetin (Huangqi and Jiangxiang, main peak at 31.029 min). The resulting HPLC chemoprofiles of these top bioactive compounds are illustrated in Figure 1.

### 2.2. QSYQ Improve Cardiac Function Following Myocardial Infarction

**Figure 2:**
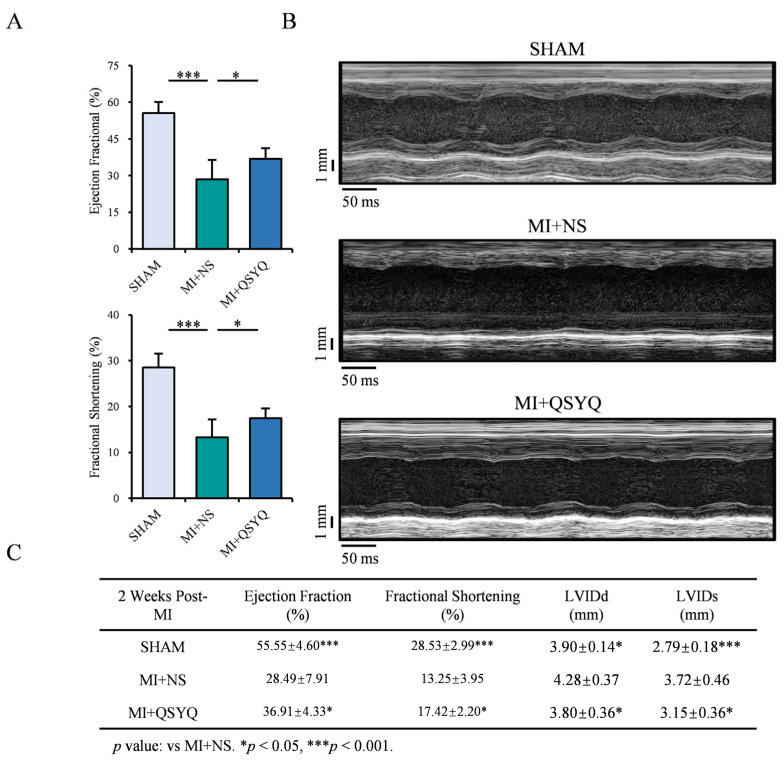
QSYQ improve cardiac function following ischemic injury. (**A**) Echocardiographic assessment of ejection fraction (EF) and fractional shortening (FS) 2 weeks post-myocardial infarction in mice administered NS or QSYQ, with additional measurements of left ventricular internal diameter at diastole (LVIDd) and left ventricular internal diameter at systole (LVIDs). *n* = 7 or more. (**B**) M-mode echocardiography 2 weeks post-myocardial infarction in mice administered with NS or QSYQ. (**C**) Cardiac function parameters in Sham, NS, and QSYQ groups at 2 weeks post-MI. *n* = 7 or more. Data are presented as mean ± SEM along with individual data points; * *p* < 0.05, *** *p* < 0.001. Abbreviations: LVIDd: left ventricular internal diameter, diastolic; LVIDs: left ventricular internal diameter, systolic.

We first investigated the potential ability of QSYQ in protecting against ischemic injury following myocardial infarction (MI). Mice underwent surgical MI and echocardiographic analysis of cardiac function after 2 weeks. There were significant reductions in the left ventricular (LV) ejection fraction (EF%) and fractional shortening (FS%) parameters in the MI + NS group versus sham-operated controls (Figure 2A), which were markedly attenuated in mice administered QSYQ. M-mode echocardiography revealed that QSYQ-administered mice had markedly improved LV wall motion compared to NS-administered mice following MI (Figure 2B). The cardiac functional parameters 2 weeks post-MI are summarized in Figure 2C. Collectively, these data indicate that QSYQ exhibit strong cardioprotective ability following myocardial infarction.

### 2.3. QSYQ Attenuate Myocardial Ischemic Injury and Decreases Cardiac Fibrosis Following Myocardial Infarction

**Figure 3:**
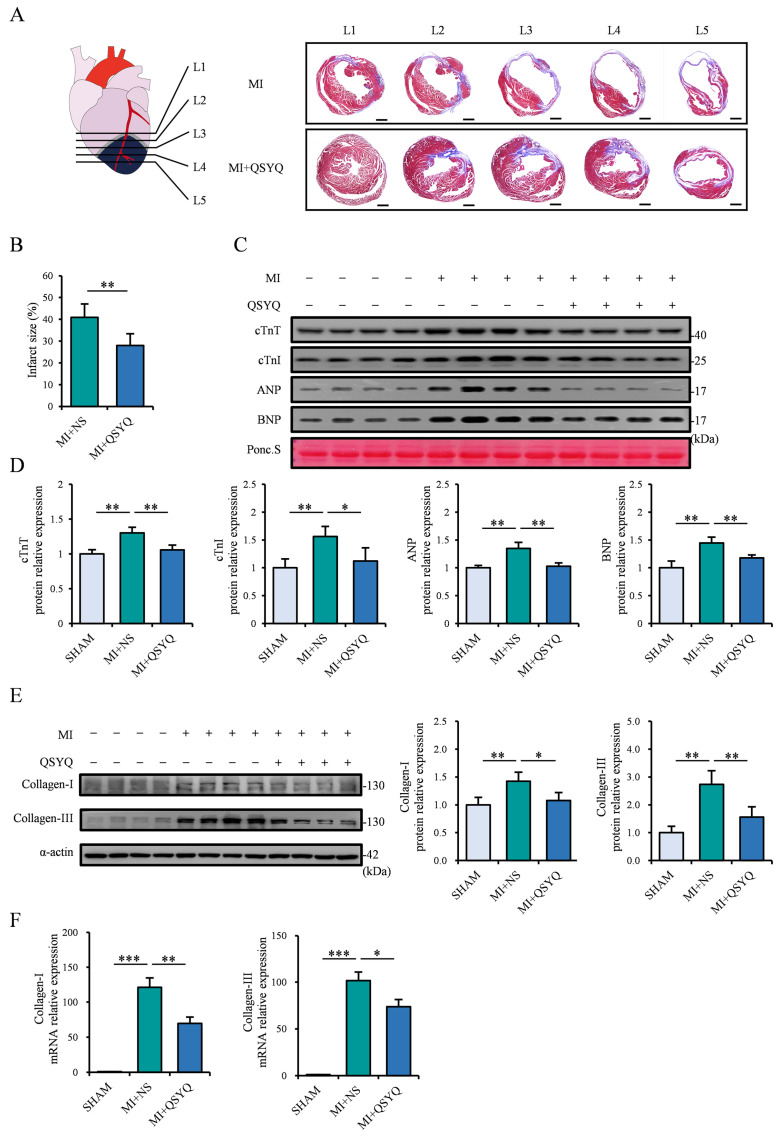
QSYQ attenuate myocardial ischemic injury and decrease cardiac fibrosis following myocardial infarction. (**A**) Cardiac schematic (**left**): pink, non-ischemic myocardium; grey, ischemic border region; blue, ischemic myocardium. Masson’s trichrome staining of hearts 4 weeks post-MI in mice administered NS or QSYQ (**right**), showing 5 transverse sections (L1–L5) from ligation site toward apex. Muscle fibers (red); fibrosis (blue). *n* = 8 or more. Scale bar: 2 mm. (**B**) Quantification of infarct size. Formula: (Mean endocardial infarct arc length at L3–L4)/(Mean left ventricular endocardial arc length at L3–L4) × 100%. (**C**,**D**) Representative immunoblots (**top**) and quantification (**bottom**) of serum cTnT, cTnI, ANP, and BNP 2 weeks post-MI. *n* = 4. (**E**) Representative immunoblots (**left**) and quantification (**right**) of collagen-I and -III protein levels 2 weeks post-MI. *n* = 4. (**F**) Collagen-I and -III mRNA levels 2 weeks post-MI. *n* = 8 or more. Data are presented as mean ± SEM along with individual data points; * *p* < 0.05, ** *p* < 0.01, *** *p* < 0.001.

We further examined the changes in infarct size 4 weeks post-MI via Masson’s trichrome staining. Transverse sections of hearts were made across five equivalent levels from the L1 surgical site to the L5 apex. QSYQ-administered mice had visibly reduced infarct size compared to NS-administered mice in all levels (Figure 3A,B). In addition, the levels of serum biomarkers of myocardial injury, including cardiac troponin T (cTnT), cardiac troponin I (cTnI), atrial natriuretic peptide (ANP), and brain natriuretic peptide (BNP) were significantly increased post-MI but markedly attenuated in QSYQ-administered mice (Figure 3C,D).

Moreover, we detected levels of collagen-I and collagen-III in the hearts post-MI, which are indicative of the degree of cardiac fibrosis following MI. Western blot and real-time PCR analyses showed that the respective protein and mRNA levels of collagen-I and -III were significantly increased post-MI but were markedly attenuated in QSYQ-administered mice (Figure 3E,F). These results collectively demonstrate the robust ability of QSYQ in protecting against cardiac ischemic injury and myocardial fibrosis.

### 2.4. QSYQ Inhibit MI-Induced Oxidative and DNA Damage

**Figure 4:**
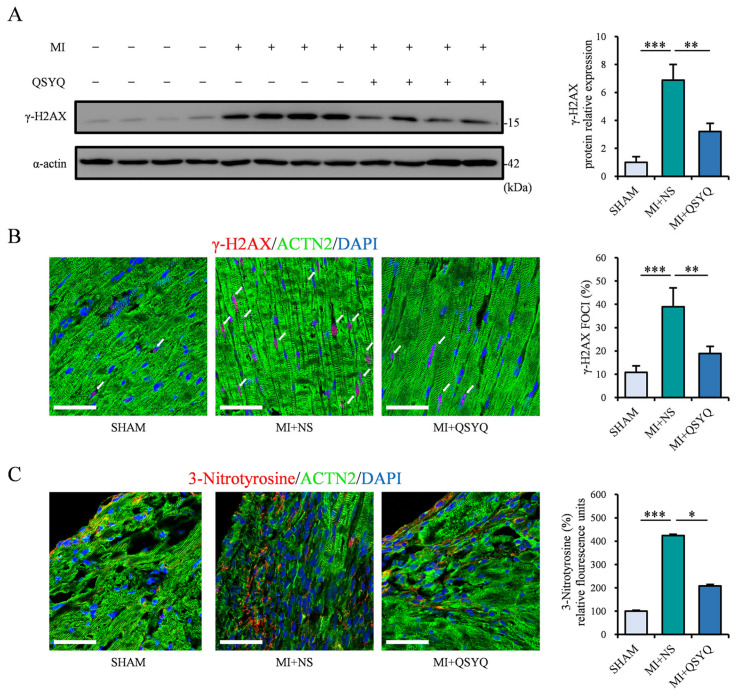
QSYQ inhibits MI-induced oxidative and DNA damage. (**A**) Representative immunoblots (**left**) and quantification (**right**) of γ-H2AX expression 2 weeks post-MI. *n* = 4. (**B**) Immunofluorescence staining (**left**) and quantification (**right**) of γ-H2AX foci (red, white arrows), α-actinin 2 (ACTN2, green), and DAPI (blue). *n* = 3. Scale bar: 50 µm. (**C**) Immunofluorescence staining (**left**) and quantification (**right**) of 3-nitrotyrosine (red), α-actinin 2 (ACTN2, green), DAPI (blue), and colocalization of 3-nitrotyrosine and ACTN2 (yellow). *n* = 3. Scale bar: 50 µm. Data are presented as mean ± SEM along with individual data points; * *p* < 0.05, ** *p* < 0.01, *** *p* < 0.001.

Next, we investigated the degree of oxidative stress and DNA damage, the key outcomes following MI. The level of γ-H2AX, a robust marker of DNA damage occurrence, was significantly elevated in the ischemic myocardium of mice 2 weeks post-MI but significantly reduced in QSYQ-administered mice, as observed via Western blot and immunofluorescence staining (Figure 4A,B). In addition, we examined the levels of 3-nitrotyrosine, which reflect the amount of protein tyrosine nitration, as a direct outcome of oxidative stress. Notably, 3-nitrotyrosine was significantly elevated in the ischemic myocardium 2 weeks post-MI, which was also markedly decreased in QSYQ-administered mice (Figure 4C). Collectively, these results indicate that QSYQ can strongly protect against myocardial-infarction-induced oxidative stress and DNA damage.

### 2.5. QSYQ Protect Against MI Damage via Activating SIRT3/FOXO3a Antioxidant Pathway

**Figure 5:**
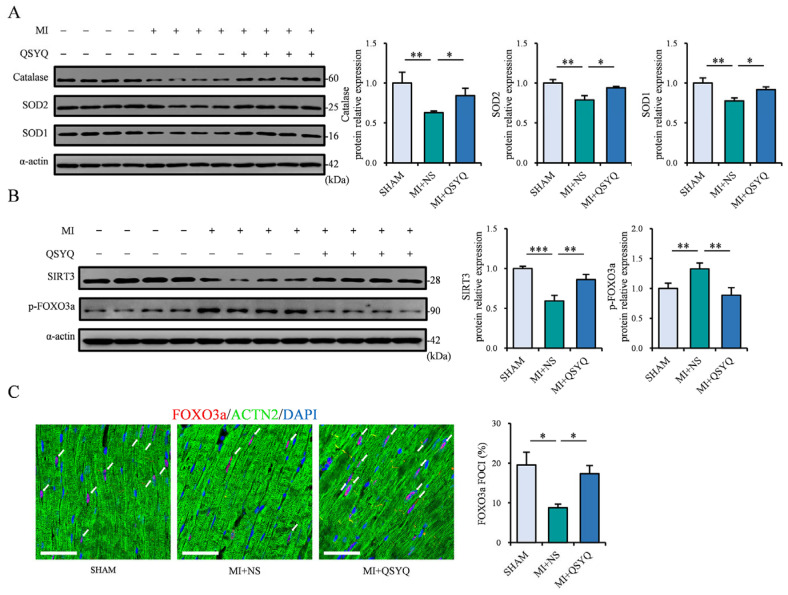
QSYQ activate the SIRT3/FOXO3a antioxidant pathway. (**A**) Representative immunoblots (**left**) and quantification (**right**) of catalase, SOD1, and SOD2 2 weeks post-MI. *n* = 4. (**B**) Representative immunoblots (**left**) and quantification (**right**) of SIRT3 and p-FOXO3a 2 weeks post-MI. *n* = 4. (**C**) Immunofluorescence staining (**left**) and quantification (**right**) of nuclear FOXO3a-positive foci (red, white arrows), α-actinin 2 (ACTN2, green), and DAPI (blue). *n* = 3. Scale bar: 50 µm. Data are presented as mean ± SEM along with individual data points; * *p* < 0.05, ** *p* < 0.01, *** *p* < 0.001.

In order to determine the underlying mechanisms of QSYQ in protecting against oxidative damage following myocardial ischemia, we examined the activation of SIRT3/FOXO3a signaling pathway, which is critically involved in antioxidant defense and redox homeostasis following MI. Western blot analysis revealed that the levels of key antioxidant enzymes, catalase, SOD1, and SOD2, were significantly decreased in the ischemic myocardium 2 weeks post-MI (Figure 5A). Notably, mice administered QSYQ significantly prevented the MI-induced decrease in these antioxidant proteins (Figure 5A), suggesting that its ability to prevent MI-induced oxidative damage is achieved via upregulating these antioxidant proteins.

In addition, the level of SIRT3 was significantly decreased in the ischemic myocardium post-MI, while the level of FOXO3a phosphorylation was upregulated, indicative of cytoplasmic retention (Figure 5B). Supporting this finding, immunofluorescence staining showed a decrease in the nuclear localization of FOXO3a following MI (Figure 5C). Notably, QSYQ administration prevented both the MI-induced SIRT3 reduction and increase in FOXO3a phosphorylation (Figure 5B) and markedly increased the nuclear localization of FOXO3a in the ischemic myocardium (Figure 5C). Collectively, these data demonstrate that QSYQ prevents oxidative ischemic damage following MI via activating the SIRT3/FOXO3a pathway and promoting the activity of downstream antioxidant proteins.

### 2.6. Administration of a Selective SIRT3 Inhibitor (3-TYP) Abolishes QSYQ Cardioprotection Post-MI

**Figure 6:**
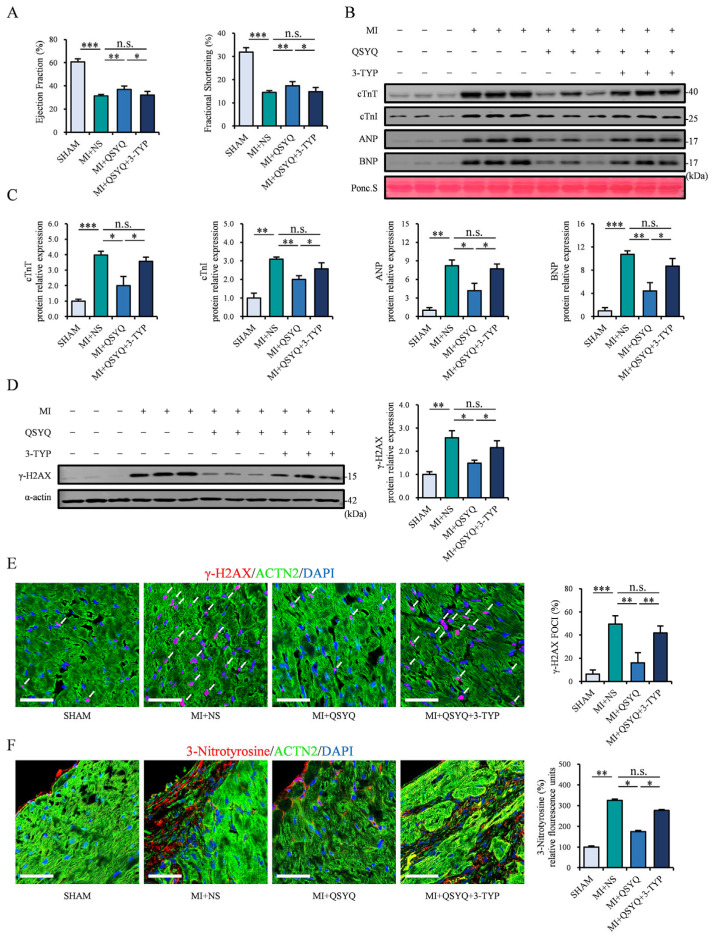
Administration of a selective SIRT3 inhibitor (3-TYP) abolishes QSYQ cardioprotection in MI. (**A**) Ejection fraction (EF) and fractional shortening (FS) in mice administered NS, QSYQ, or QSYQ + 3-TYP at 2 weeks post-MI. *n* = 6. (**B**,**C**) Representative immunoblots (**B**) and quantification (**C**) of serum cTnT, cTnI, ANP, and BNP 2 weeks post-MI. *n* = 3. (**D**) Representative immunoblots (**left**) and quantification (**right**) of cardiac γ-H2AX. *n* = 3. (**E**) Immunofluorescence staining (**left**) and quantification (**right**) of γ-H2AX foci (red, white arrows), α-actinin 2 (ACTN2, green), and DAPI (blue). *n* = 3. Scale bar: 50 µm. (**F**) Immunofluorescence staining (**left**) and quantification (**right**) of 3-nitrotyrosine (red), α-actinin 2 (ACTN2, green), DAPI (blue), and colocalization of 3-nitrotyrosine and ACTN2 (yellow). *n* = 3. Scale bar: 50 µm. Data are presented as mean ± SEM with superimposed individual data points; n.s. *p* > 0.05, * *p* < 0.05, ** *p* < 0.01, *** *p* < 0.001.

In order to confirm that the protective ability of QSYQ was achieved via the activation of the SIRT3/FOXO3a pathway, we further administered a selective SIRT3 inhibitor, 3-(1H-1,2,3-triazol-4-yl) pyridine (3-TYP), in mice starting from 1 week prior to MI. The echocardiography results showed that mice that received co-administration of QSYQ and 3-TYP no longer exhibited an increase in cardiac function 2 weeks post-MI (Figure 6A). Western blot analysis revealed that the QSYQ + 3-TYP co-administration group had similarly high levels of serum cardiac injury markers (cTnT, cTnI, ANP, BNP) compared to the NS group post-MI (Figure 6B,C). Moreover, the levels of DNA and oxidative damage markers, γ-H2AX protein and 3-nitrotyrosine, were no longer decreased in the QSYQ + 3-TYP group (Figure 6D–F), demonstrating that the protective effects of QSYQ in myocardial ischemic injury require the activation of SIRT3.

**Figure 7:**
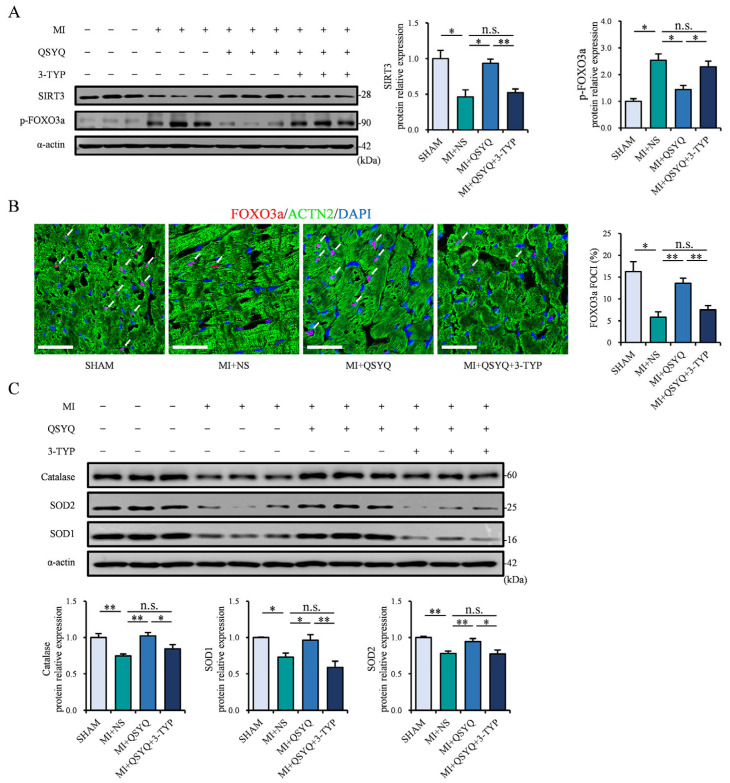
3-TYP significantly blocks QSYQ-induced SIRT3/FOXO3a pathway activation. (**A**) Representative immunoblots (**left**) and quantification (**right**) of SIRT3 and p-FOXO3a 2 weeks post-MI. *n* = 3. (**B**) Immunofluorescence staining and quantification of nuclear FOXO3a-positive foci (red, white arrows), α-actinin 2 (ACTN2, green), and DAPI (blue). *n* = 3. Scale bar: 50 µm. (**C**) Representative immunoblots (**top**) and quantification (**bottom**) of catalase, SOD1, and SOD2. *n* = 3. Data are presented as mean ± SEM with superimposed individual data points; n.s. *p* > 0.05, * *p* < 0.05, ** *p* < 0.01.

Western blot analysis further demonstrated that SIRT3 could no longer be activated and the subsequent FOXO3 nuclear localization was not induced post-MI in the QSYQ + 3-TYP co-administration group (Figure 7A,B). Moreover, the levels of downstream antioxidant enzymes catalase, SOD1, and SOD2 were suppressed in the ischemic myocardium in the QSYQ + 3-TYP group (Figure 7C), demonstrating that QSYQ-induced activity of these antioxidant proteins is mediated by the SIRT3/FOXO3a signaling pathway. Taken together, these data demonstrate that QSYQ protects against ischemic injury following myocardial infarction via activation of SIRT3/FOXO3a pathway.

## 3. Discussion

A major outcome of myocardial infarction is an increased generation of reactive oxygen species (ROS), which is the main cause of oxidative stress in the ischemic myocardium. Intracellular ROS levels are tightly regulated by an antioxidant defense network, encompassing various enzymatic systems such as glutathione peroxidase/glutathione reductase, catalase, superoxide dismutase, and peroxiredoxin/thioredoxin. MI disrupts the homeostatic balance between the amount of ROS versus antioxidant enzymes, which results in oxidative stress, DNA damage occurrence, and ultimately cardiomyocyte death [20,21]. Because damage to cardiomyocytes is irreversible, discovering novel and effective therapeutic strategies that can augment the production of key antioxidant enzymes following MI may be critical [22,23]. Among these enzymatic systems, SOD and catalase constitute the primary intracellular antioxidant defense mechanism that effectively converts ROS into water and oxygen. This enzymatic conversion represents the core mechanism of antioxidant defense against ROS toxicity [24]. Therefore, our current study, which demonstrates the ability of QSYQ in robustly activating the key antioxidant enzymes catalase and SOD post-MI, implicates the therapeutic potential of QSYQ in the treatment of myocardial infarction.

Antioxidant enzyme levels are regulated by multiple signaling pathways, including the KEAP1/NRF2 [25], AMPK/mTOR-TFEB [26], PPARγ/PGC-1α [27], and SIRT3/FOXO3a signaling pathways [12]. Among these pathways, SIRT3/FOXO3a signaling plays a critical role in maintaining mitochondrial homeostasis and protecting against pathological oxidative stress. SIRT3 is a mitochondrial deacetylase that is highly expressed in various tissues including the heart and facilitates the nuclear transport of the transcriptional factor FOXO3a. Subsequently, FOXO3a triggers the activation of core antioxidant enzymes and clearance of ROS, thereby conferring mitochondrial redox homeostasis against oxidative injury such as in ischemic heart disease [12,28,29,30]. Thus, discovering drugs that can enhance activation of SIRT3/FOXO3a is crucial for mitigating the damage caused by oxidative stress following MI. Our current study found that QSYQ administration significantly increased the levels of SIRT3 following MI, enhanced FOXO3a nuclear translocation, resulting in the activation of key antioxidant enzymes SOD1, SOD2, and catalase. Therefore, drugs such as QSYQ that can decrease myocardial injury post-MI while also robustly activating SIRT3 and downstream ROS neutralizing enzymes are urgently needed in the clinical setting.

Previous clinical studies have demonstrated the positive cardiovascular benefits of QSYQ, including in randomized double-blind clinical trials [31] as well as in meta-analysis of numerous trials, confirming that QSYQ improve outcomes in heart failure patients [32,33]. Pharmacological studies have demonstrated that QSYQ ameliorated post-myocardial infarction cardiac dysfunction and ventricular remodeling via suppression of the TGF-β1/Smad3 fibrotic axis and reducing collagen deposition [34]. Furthermore, QSYQ ameliorated post-myocardial infarction injury and cardiac fibrosis via the modulation of Nrf2-PITX2 signaling [35] and cAMP/Rap1 pathways [36]. Our current study is the first to demonstrate that QSYQ prevent oxidative-stress-induced myocardial damage following MI via the specific activation of the SIRT3/FOXO3a pathway and downstream antioxidant enzymes, extending the current research regarding the beneficial role of QSQY in ischemic heart disease. A recent proteomics analysis showed that QSQY were protective in ischemia-induced heart failure via suppressing mitochondrial fission, a process that is closely linked to oxidative stress regulation [37], thus corroborating the findings from our current study.

There are several limitations to our study. Firstly, our current study utilized a murine model of myocardial infarction; however, due to the inherent species difference between mouse and human pathophysiology, additional studies in humans are needed to better confirm QSQY’s cardioprotective effects in a clinical setting. Secondly, due to the pharmacological complexity of a multi-component formulation such as QSYQ, it is challenging to attribute the beneficial effects to any one specific compound; rather, they may be due to the synergistic action of multiple components. Finally, although our study utilized a selective SIRT3 inhibitor 3-TYP to support our findings, additional experiments utilizing gene knockout mice are needed to provide better mechanistic support for the causal role of the SIRT3/FOXO3a axis. Despite these limitations, our findings provide novel insights into the cardioprotective mechanisms of QSQY following MI and a theoretical basis for the potential use of QSQY in the clinical treatment of myocardial infarction.

## 4. Materials and Methods

### 4.1. Preparation of QSYQ

Qishen Yiqi dropping pills (QSYQ) formulation (National Medicine Permit No. Z20113048) was provided by Tasly Pharmaceutical Group Co., Ltd. (Tianjin, China). The constituents of Qishen Yiqi dropping pills consisted of Mongolian Milkvetch (*Astragalus membranaceus* (Fisch.) Bge. var. mongholicus (Bge.) Hsiao), Red-rooted Sage (*Salvia miltiorrhiza* Bunge), Notoginseng (*Panax notoginseng* (Burk.) F.H.Chen), and Rosewood (*Dalbergia odorifera* T.Chen), mixed in a ratio of 150:75:15:1. For extract preparation, QSYQ was processed into a fine powder and sieved (180 µm). The resulting powder was suspended in sterile normal saline at 100 mg/mL, followed by filtration through a 0.22 µm membrane and storage at −20 °C. Mice were administered Qishen Yiqi dropping pills via intragastric gavage at a dose of 0.54 g·kg^−1^·d^−1^.

### 4.2. High-Performance Liquid Chromatography–Mass Spectrometry (HPLC-MS/MS)

HPLC-MS/MS fingerprinting analysis was used for identifying the chemical profiles of the top bioactive compounds contained within QSYQ. Briefly, 0.3 g of QSYQ powder extract was made up to a total concentration of 1 mg/mL, and standards were injected into the HPLC-MS/MS system (LC-30A, Shimadzu, Kyoto, Japan) and then subsequently separated on a C18 ODS column (1.8 μm, 2.1 × 100 mm) via gradient elution. Then, 0.2% 2-sulfobenzoic acid hydrate (A) and acetonitrile (B) were used as the mobile phase, and the gradient elution procedure was as follows: 0 min, A:B = 97:5; 0.01 min, A:B = 75:30; 37 min, A:B = 95:5; 37.1 min, with a flow rate of 0.5 mL/min. The resulting HPLC chemoprofiles of top bioactive compounds were determined according to their retention times with their respective intensity peaks.

### 4.3. Animal Model

Wild-type C57BL/6J mice (male, ten weeks old) were used in models of myocardial infarction. Mice were acclimatized and maintained under specific pathogen-free laboratory conditions including a 12 h light/dark cycle, controlled temperature (22 ± 2 °C), and access to standard rodent chow and drinking water ad libitum. All procedures complied with ethical guidelines and were approved by the Laboratory Animal Care and Use Committee of Fujian University of Traditional Chinese Medicine (Fujian, China; approval No.: FJTCM-IACUC 2023058, approval date: 8 May 2023). Surgical models of myocardial infarction were established according to standard protocol, as previously described [38,39]. Briefly, mice were first anesthetized via sodium pentobarbital (intraperitoneal, 50 mg kg^−1^), and the left anterior descending coronary artery (LAD) was permanently ligated. Sham-operated mice underwent the same surgical procedure without ligation. All surgical procedures and subsequent analyses were performed by an investigator blinded to the experimental groups. Mice were randomly allocated into three groups: (1) sham operation group; (2) MI + NS group; (3) MI + QSYQ group (0.54 g·kg^−1^·d^−1^), with a group size of *n* = 8 or more. A three-week dosing regimen was implemented as follows: mice received intragastric administration from one week prior to surgery until two weeks post-MI induction for a total of three weeks. Mice in sham-operated and MI + NS groups received an equal volume of sterile saline daily, while the MI + QSYQ group received QSYQ (0.54 g kg^−1^ per day), divided into two equal doses administered in the morning and evening. A separate experiment using the inhibitor 3-TYP (HY-108331, MedChemExpress, Monmouth Junction, NJ, USA) was grouped as follows: (1) sham operation group; (2) MI + NS group; (3) MI + QSYQ group (0.54 g·kg^−1^·d^−1^); (4) MI + QSYQ + 3-TYP group (3-TYP: 50 mg kg^−1^, every two days), with a group size of *n* = 6 or more. In this experiment, QSYQ was administered following the same regimen as described above, while mice in the 3-TYP group received intraperitoneal injection of TYP (50 mg kg^−1^, once every two days).

### 4.4. Echocardiography Assessment

Cardiac function was assessed by a blinded investigator following standardized protocols. Briefly, mice were anesthetized via continuous inhalation of isoflurane (Sigma Aldrich, St. Louis, MO, USA) delivered through a vaporizer (EZ Anesthesia, Cumming, GA, USA). Mouse body temperature was maintained at 37 °C, and heart rates were stabilized at approximately 450 beats per minute prior to measurements. Two-dimensional M-mode images were acquired in parasternal long-axis view using echocardiography imaging system (Vevo 2100 Imaging System, VisualSonics, Toronto, ON, Canada) for determination of cardiac function parameters including left ventricular ejection fraction (EF) and fractional shortening (FS).

### 4.5. Serum Collection

Serum was collected post-MI via mouse tail vein, centrifuged (3000 rpm, 10 min) and stored at −80 °C. Subsequently, levels of serum biomarkers (cTnT, cTnI, ANP, BNP) were assessed via Western blot.

### 4.6. Western Blot Analysis

Western blot analyses were conducted according to standard protocol. Briefly, total protein was extracted from mouse heart tissues using RIPA lysis buffer containing protease and phosphatase inhibitors. Equal amounts of protein underwent SDS-PAGE separation and subsequent transfer onto PVDF membranes (Merck Millipore, Burlington, MA, USA). Membranes were blocked with 5% non-fat dry milk, then incubated with primary antibodies at 4 °C overnight. Subsequently, membranes were incubated with the respective HRP-conjugated secondary antibodies and protein bands were detected using enhanced chemiluminescence (ECL). Relative expression levels were quantified through densitometric analysis with ImageJ software (version 1.53s). The following antibodies were used: ANP (ab225844), BNP (ab236101), cTnI (ab47003), cTnT (ab8295), γ-H2AX (ab81299), FOXO3a (ab23683), p-FOXO3a (ab154786), and catalase (ab209211) were purchased from Abcam, Cambridge, UK; collagen-I (14695-1-AP), collagen-III (22734-1-AP), SIRT3 (10099-1-AP), SOD1 (10269-1-AP), SOD2 (24127-1-AP), and α-actin (23660-1-AP) were purchased from Proteintech, Rosemont, IL, USA.

### 4.7. Quantitative Real-Time Polymerase Chain Reaction (qRT-PCR)

Real-time PCR analysis was used to determine relative mRNA expression in heart tissues and was performed according to standard protocol. Briefly, total RNA was extracted using TRIzol reagent (Takara Biotechnology, Tokyo, Japan), and the cDNA was synthesized using a cDNA Synthesis kit (Takara Biotechnology, Tokyo, Japan). Quantitative PCR amplification and detection were then carried out using SYBR Green Master Mix (Applied Biosystems, Carlsbad, CA, USA). The primer sequences for collagen-I were 5′-ATGGATTCCCGTTCGAGTAC-3′ (forward) and 5′-TCAGCTGGATAGCGACATCG-3′ (reverse). The primer sequences for collagen-III were 5′-CGTAGATGAATTGGGATGCA-3′ (forward) and 5′-ACATGGTTCTGGCTTCCAG-3′ (reverse). The primer sequences for catalase were 5′-GGAGGCGGGAACCCAATAG-3′ (forward) and 5′-GTGTGCCATCTCGTCAGTGAA-3′ (reverse). The primer sequences for GAPDH were 5′-TGGCCTTCCGTGTTCCTAC-3′ (forward) and 5′-GAGTTGCTGTTGAAGTCGCA-3′ (reverse). GAPDH was used as reference gene for determination of relative gene expressions. Relative fold changes were analyzed using the comparative Ct (ΔΔCt) method and normalized to the sham-operated control group.

### 4.8. Histology

Histological analyses were performed to evaluate the degree of myocardial fibrosis according to standard protocol. Briefly, cardiac tissues were fixed in 4% paraformaldehyde overnight, then dehydrated, embedded in paraffin, and cut into 5 µm sections. Masson’s trichrome staining was used to determine the extent of myocardial fibrosis following MI.

### 4.9. Immunofluorescence

Cardiac tissue sections were processed for immunofluorescence following standard protocol. Briefly, samples were fixed in 4% ice-cold paraformaldehyde (PFA; Sigma Aldrich, St. Louis, MO, USA), permeabilized with 0.25% Triton X-100 (Sigma Aldrich, St. Louis, MO, USA), and blocked in 5% BSA (Roche, Basel, Switzerland). Sections were incubated overnight at 4 °C with primary antibodies: 3-nitrotyrosine (ab110282, Abcam, Cambridge, UK), ACTN2 (14221-1-AP, Proteintech, Rosemont, IL, USA), ACTN2 (A7811, Merck Millipore, Darmstadt, Germany), then the respective secondary antibodies (Abcam, Cambridge, UK), and mounted in DAPI-containing anti-fade medium (Beyotime Biotechnology, Shanghai, China). Slides were imaged using Zeiss LSM 710 confocal microscope (Oberkochen, Germany).

### 4.10. Statistical Analysis

All statistical analyses were performed using SPSS 26.0 software (Chicago, IL, USA). Data are expressed as the mean ± standard error of the mean (SEM). Shapiro–Wilk tests for normality were performed to ensure outcomes with normal distribution, and, thereafter, comparisons of means were performed using independent samples *t*-test (between two groups) or one-way ANOVA with Fisher’s LSD post hoc analysis (between three and more groups). For all analyses, *p* < 0.05 was considered statistically significant.

## Data Availability

The datasets used and/or analyzed during the current study are available from the corresponding author on reasonable request.

## Abbreviations

The following abbreviations are used in this manuscript:

3-TYP	3-(1H-1,2,3-triazol-4-yl) pyridine
ANP	Atrial Natriuretic Peptide
BNP	Brain Natriuretic Peptide
cTnI	Cardiac Troponin I
cTnT	Cardiac Troponin T
EF	Ejection Fraction
FS	Fractional Shortening
LAD	Left Anterior Descending (coronary artery)
LV	Left Ventricular
MI	Myocardial Infarction
QSYQ	Qishen Yiqi Dropping Pills
ROS	Reactive Oxygen Species
SOD	Superoxide Dismutase
